# Inflammation and Oxidative Stress in Frailty and Metabolic Syndromes—Two Sides of the Same Coin

**DOI:** 10.3390/metabo13040475

**Published:** 2023-03-26

**Authors:** Sylwia Dzięgielewska-Gęsiak, Małgorzata Muc-Wierzgoń

**Affiliations:** Department of Internal Medicine Prevention, Medical University of Silesia in Katowice, 40-055 Katowice, Poland

**Keywords:** metabolic syndrome, frailty syndrome, inflammatory markers, oxidant–antioxidant balance

## Abstract

In developed countries, aging is often seen as typical, but it is made complicated by many disorders and co-morbidities. Insulin resistance seems to be an underlying pathomechanism in frailty and metabolic syndromes. The decline in insulin sensitivity leads to changes in the oxidant–antioxidant balance and an accelerated inflammatory response, especially by adipocytes and macrophages in adipose tissue, as well as muscle mass density. Thus, in the pathophysiology of syndemic disorders—the metabolic syndrome and frailty syndrome—an extremely important role may be played by increased oxidative stress and pro-inflammatory state. Papers included in this review explored available full texts and the reference lists of relevant studies from the last 20 years, before the end of 2022; we also investigated the PubMed and Google Scholar electronic databases. The online resources describing an elderly population (≥65 years old) published as full texts were searched for the following terms: “oxidative stress and/or inflammation”, “frailty and/or metabolic syndrome”. Then, all resources were analyzed and narratively described in the context of oxidative stress and/or inflammation markers which underlie pathomechanisms of frailty and/or metabolic syndromes in elderly patients. So far, different metabolic pathways discussed in this review show that a similar pathogenesis underlies the development of the metabolic as well as frailty syndromes in the context of increased oxidative stress and acceleration of inflammation. Thus, we argue that the syndemia of the syndromes represents two sides of the same coin.

## 1. Introduction

In recent years, there are alarming data showing a worldwide increase in mortality in the elderly group, which is not only the result of deaths due to the COVID-19 pandemic, but also most common age-related diseases [1,2,3,4]. In developed countries, aging is often seen as typical, it is associated with dysfunctions resulting from an incorrect lifestyle (poor or bad balanced diet, lack of physical activity) [5,6,7,8]. Incorrect diet and sedentary lifestyle are risk factors for overweight and obesity, decrease in muscle mass density and elevated blood pressure as well result in biochemical changes such as insulin-resistance, carbohydrate metabolism, dyslipidemia and dysproteinemia [9,10]. Through the human lifespan, unfavorable biochemical phenomena accumulate, including increased inflammation and the progression of oxidative stress, which result in the manifestation of clinical dysfunctions and, as a result, premature death [11,12]. Thus, aging is complicated by disorders such as decreased insulin sensitivity, hyperglycemia, hyperlipidemia or civilization diseases such as diabetes, hypertension and cardiovascular diseases. Gathered clinical and metabolic conditions are seen in the metabolic syndrome [13,14,15]. The main pathomechanism in the metabolic syndrome is insulin resistance. With a growing silver population together with body changes, metabolic syndrome patients are increasing in number. What is more, with aging, interpersonal relationships deteriorate—resulting from multiple illnesses, social isolation or financial conditions. Worse functioning in the bio–psycho–social sphere leads to geriatric syndrome—an epidemic of the twenty-first century—frailty syndrome [16,17]. Linda Fried’s concept sees frailty syndrome as a pathophysiologically distinct process. It results from the dysfunction of biological mechanisms and primary physiological processes in the elderly [18], while Rockwood et al. argue that frailty syndrome is a consequence of the overlapping of many deficits and diseases [19].

So far, the literature has assessed the metabolic syndrome and frailty syndrome separately, while simultaneously describing similar pathomechanisms leading to the formation of these syndromes, including hormonal disorders, the acceleration of inflammatory processes and an oxidant–antioxidant imbalance [20,21].

With advancing age, there are changes in the endocrine system. An increase in the concentration of glucocorticoids, which are known to play a role in the inflammation processes, is observed [22]. In addition, the concentrations of sex hormones are reduced—both testosterone in men and estradiol in women [23,24]. Moreover, the secretion of growth hormone decreases, and this results in a decrease in the synthesis of insulin-like growth factor 1 (IGF1) affecting muscle mass and function [25]. The IGF1 also has a protective effect against increased oxidative stress and is a marker of frailty status in the elderly [26].

Enhanced inflammation, increased reactive oxygen spaces (ROS) production and faint antioxidant defense systems are responsible for the improper synthesis, secretion and action of insulin leading to insulin resistance [27,28,29]. Insulin resistance, defined as a reduced sensitivity of body cells to insulin, is a common disorder among patients with metabolic syndrome, as well as with frailty syndrome. It coexists with the disturbance of body mass and worsening of glucose tolerance and an increased risk of developing type 2 diabetes [30]. The decline in insulin sensitivity is the main reason for the development of hyperglycemia. This is the basis and the main cause of the microangiopathies which lead to ocular, kidney and neural pathology, and atherosclerosis, which results in cardiovascular complications [31,32,33,34]. What is more, insulin resistance has an influence on lipids metabolism and protein synthesis. Moreover, lower insulin sensitivity causes an imbalance toward muscle mass density, relative handgrip force and decreased level of physical activity with an outcome of sarcopenia and thus leads to the clinical face of frailty syndrome [35]. In the pathophysiology of this phenomenon, changes related to the increased oxidative stress and inflammatory response in adipose tissue deserve special attention, as a result of which adipocytes and macrophages produce ROS and pro-inflammatory adipocytokines and thus decrease insulin sensitivity [36]. Immunometabolic adipose tissue changes are related to the development of systemic chronic, low-grade inflammation and increased systemic oxidative stress [37]. With the development of such changes, clinical complications and comorbidities also develop [38,39].

Thus the aim of this narrative review is to pay attention to the interrelationships between the impact of inflammation, oxidative stress markers and various metabolic pathways in the development of frailty and metabolic syndromes in elderly individuals, which underlie the pathogenesis of these syndromes.

**Figure 1:**
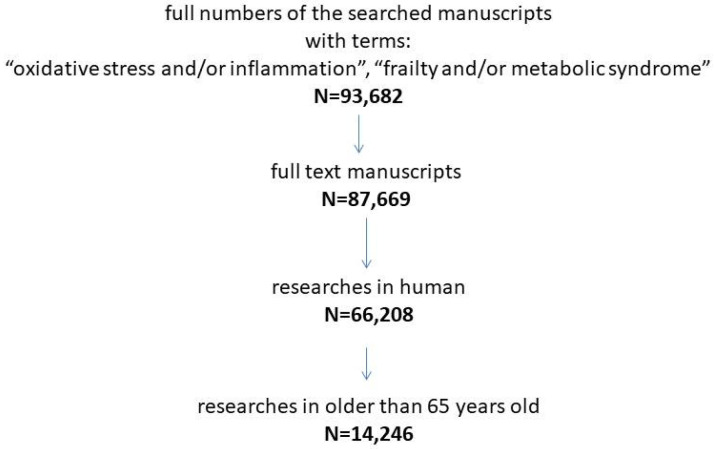
The flow chart describes the search strategy of the articles.

For this purpose, we explored the PubMed and Google Scholar electronic databases (full numbers of the searched manuscripts were *n* = 93,682). However, the inclusion criteria of the online resources described were systematically decreasing with adding further filters: only human researches (*n* = 87,669), elderly population (≥65 years old) (*n* = 14,246). We searched published manuscripts as full texts from 2002 to the end of 2022 for the following terms: “oxidative stress and/or inflammation”, “frailty and/or metabolic syndrome”. This was supplemented with manually searches for possible missing articles. Figure 1 describes the search strategy of the papers included in the article.

The review summarizes the most up-to-date articles that were applicable to this study, which included systematic reviews, meta-analyses, qualitative and quantitative studies and opinion pieces. Then, all resources were analyzed and narratively described in the context of oxidative stress and/or inflammation markers which underlie pathomechanisms of frailty and/or metabolic syndromes in elderly individuals.

## 2. Theories of Aging, Oxidative Stress and Inflammaging

Many mechanisms of aging have been described thus far [40,41,42]. Aging is perceived either: as a pre-programmed process (programmed death theories—understood as a decrease in repair processes, a decrease in the body’s ability to recreate cells and the loss of reproductive capacity); or as a result of the accumulation of damage to cells, tissues and/or organs (error accumulation theories—it is caused by the influence of external environmental factors: biological, chemical and physical, causing errors in the genetic material, which accumulate in subsequent generations of cells). Gerontological hypotheses associate the aging of the organism with changes taking place in the extracellular matrix, cells capable to divide, and cells which finished their division processes [43,44,45]. Aging is affected by many factors that have not yet been thoroughly explained, which means that the small amount of data collected is the reason for numerous theories relating to the aging process.

As early as 1956, Denham Harman hypothesized that the cause of aging was cell damage by free radicals. In the free radical theory of aging, the free radicals constantly attack genetic material and other molecules, causing damage, despite the repair processes by the antioxidant systems - the existing enzymes that break down free radicals [46]. This damage accumulates in cell structures, directly affecting their functioning, accelerating aging and shortening the lifespan of the organism [47]. Studies on the aging of the body have not lost their relevance and have been widely discussed in the last decade [48,49,50].

The largest source of free radicals, e.g., superoxide anion radical, are in the cells of mitochondria. The higher the intensity of cellular respiration, the more is produced. The free radical theory of aging was extended by stating that aging can be slowed down by a diet rich in exogenous antioxidants (vitamins, coenzyme Q, melatonin, curcumin, resveratrol and other polyphenols); however, the results of research on extending human life in the context of the free radical theory of aging are unsatisfactory and debatable [51]. What is more, obtained clinical results in the healthy aged population showed discrepancies. Kozakiewicz and colleagues described a decrease in crucial activity of antioxidative enzymes with the simultaneous intensity of lipids peroxidation [52], while Vázquez-Lorente et al. argued that in a postmenopausal population, antioxidant parameter values showed no changes with aging [53], but the activity of total antioxidant capacity were affected by body composition and directly related to plasma proteins [54]. Differences in the results of the researchers may be explained by the selection of different populations, age ranges or genders, co-morbidities or even no disease existence in investigated age groups. Additionally, the explanation of the differences in the investigations may be the caloric restriction theory—the most proven theory of effective aging. This theory assumes a significant reduction in the amount of food intake, while maintaining the full valency of its nutrients [55]. Limiting the intake of nutrients forces a reduction in the intensity of metabolism, and thus, the production of reactive oxygen species is reduced. A smaller amount of free radicals means less damage to the genetic material and accumulation of errors in the next generations of cells. And, thus, much lower oxidative stress, as well as decreased inflammatory processes [47]. However, in the elderly population, the systematic review of randomized control trials,, regarding nutritional supplements, showed positive effects in terms of mitochondrial antioxidant capacity and decreased oxidative stress as well as volume of mitochondrial transcriptome [56]. Therefore, in the elderly population, we still need more complex and multi-level investigations due to the oxidative stress and accelerated inflammation.

At this point, the complex and systemic nature of aging was characterized by the concept of immunosenescence and inflammaging [57]. As the body ages, the immune system declines in terms of its ability to trigger the effective production of cells and antibodies against infections. From an evolutionary perspective, immunosenescence can be looked upon as an adaptive process. Moreover, the immune system, alongside aging, develops low-grade, chronic inflammation, which results in systemic damage but not yet clinically detectable disease [58]. Low-grade inflammation is a contributor of gut microbiota variations, and its derived products and may trigger metabolic syndrome [59]. Furthermore, if chronic inflammation processes continue, this increases the production of reactive oxygen spaces and may disrupt the total antioxidant capacity of the body. For this cross talk between inflammatory and oxidative stress mediators processes, Valacchi et al. have proposed the term “oxinflammation” [60]. Thus, dysfunction in inflammatory response can result in the destruction of the oxidant–antioxidant balance in the body and lead to overt pathological dysfunction such as metabolic and/or frailty syndromes.

## 3. Oxidative Stress and Inflammation Markers in Elderly Individuals with Metabolic Syndrome

Increased oxidative stress and accelerated inflammation, in the context of metabolic syndrome, understood as the coexistence of visceral obesity, atherogenic dyslipidemia, glycemic disorders and clinically recognized high blood pressure, has many possible sources. The already-discovered mechanisms are explained on the molecular level or go through proteomics toward a metabolomics explanation.

Karaman et al. suggested that DNA damage contributes to the pathogenesis of metabolic syndrome [61]. The authors demonstrated an elevated concentration of malondialdehyde (MDA) as the end-product of lipid peroxidation—the oxidative stress marker—in metabolic syndrome patients, and they found a significantly higher length of comet tails and micronucleus frequency, which indicates DNA damage. Since then, it has been established that increased oxidative stress in metabolic syndrome patients leads to DNA damage.

In chronic hyperglycemia-induced complications, results noted by Yan et al. indicated that sodium butyrate—microbiota-derived metabolite—may decrease oxidative stress and inflammation via a PI3K-dependent autophagy pathway [62]. Additionally, it was shown that gut microbiota directly affect adipose tissue macrophages by Toll-like receptor 4 to induce adipose tissue fibrosis [63]. What is more, in many co-morbidities including metabolic syndrome, it was identified that the peroxisome proliferator-activated receptor-γ co-activator 1α (PGC-1α) improves inflammation by regulating key antioxidant gene expression [64].

It Is noteworthy that the activity of superoxide dismutase (SOD),IIch is the first antioxidant enzyme in the cell and participates in the cell’s defense against ROS, was associated with the occurrence of metabolic syndrome in elderly Mexican patients. Additionally, together with other antioxidant enzymes, glutathione peroxidase (GPx) and reduced glutathione (GSH) correlated with the components of metabolic syndrome [65]. In addition, Suriyaprom et al. found significantly lower SOD activity and vitamin C concentration and higher white blood cell counts in Thais with metabolic syndrome [66]. What is more, Liu et al. indicated that SOD activity can be an independent predictor of metabolic syndrome, as the decrease in SOD activity was found to be independent of cell aging and pro-oxidants, as well as calorie intake and dietary antioxidants [67].

Oxidative stress means an imbalance in the higher production of pro-oxidants (MDA and protein carbonyl) and decreased activity of antioxidants enzymes (catalase, SOD, GPx and GSH). According to Awadallah et al., the best predictor of oxidant–antioxidant imbalance in metabolic syndrome individuals (increased MDA and raised protein carbonyl and lower levels of antioxidant enzymes) was waist circumference even when adjusted for the age and gender [68]. Furthermore, Sakhaei et al. found that the enzyme activity of glutathione peroxidase in serum was significantly lower in patients with metabolic syndrome compared to control, negatively correlated with diastolic blood pressure and with C-reactive protein (CRP) and positively with endothelial dysfunction markers [69]. What is more, Gyawali et al. found alterations of hemorheology due to the increased chronic inflammation and exhausted antioxidant defense mechanisms in metabolic elderly participants [70]. In a metabolic syndrome population, they found significant changes in antioxidant enzymes (SOD and GSH) which linearly correlated with erythrocyte aggregation and whole blood viscosity. Simultaneously, the thrombotic marker D-dimer increased.

Sedentary lifestyle through metabolic syndrome leads to cardiovascular complication. In the context of treatment of the metabolic syndrome, a special role is played by the influence on healthy nutrition, physical activity and sleep hygiene. In elderly Koreans with metabolic syndrome, antioxidant-rich dietary intervention for four weeks resulted in a decrease in oxidized low-density lipoproteins and lipid peroxidation levels (which are oxidative stress markers) and ameliorated the central obesity, dyslipidemia, hypertension, and arterial stiffness [71]. Mendoza-Núñez et al. studied the effects of tai chi exercise on antioxidant markers in metabolic syndrome, sedentary older patients. Total antioxidant status was significantly improved—increased in those who finished tai chi program (5 days a week for 6 months, in sessions of fifty minutes) in comparison with those who did not perform exercises. However, cardiovascular parameters did not change [72]. What is more, Osali et al. showed that a diet rich in antioxidants (curcumin supplementation) together with training improved stress oxidative indices (increase in total antioxidant capacity and decrease in MDA) and inflammation markers (significant decreases in serum concentration of tumor necrosis factor α (TNF-α), interleukin 6 (IL-6) and hs-CRP and increases in serum concentrations of brain-derived neurotrophic factor (BDNF) and interleukin 10 (IL-10)) in elderly women with metabolic syndrome [73]. Additionally, in elderly individuals, short sleep duration was independently associated with increased inflammatory load (i.e., CRP, IL-6, TNF, and IFN-γ levels) [74].

Metabolic syndrome do not occur in isolation and commonly increase in the presence of other health deficits. The secondary analysis of the Beijing Longitudinal Study of Ageing showed that if the number of cardiometabolic disorders rises, then the possibility of developing frail syndrome increases to as much as 30 percent [75]. The results correspond with studies from Western countries which have shown that cardiovascular events, cancer and other co-morbidities and total mortality are associated with frailty [76,77,78,79].

## 4. Oxidative Stress and Inflammation Markers in Elderly Individuals with Frailty Syndrome

Aging reduces physical and mental body reserves and, together with adverse diseases outcomes, results in frailty syndrome [80,81,82]. Frailty syndrome observed in older adults is caused by multiorgan and multisystem incoherence due to increased vulnerability to stressors [83,84]. It leverages muscle mass strength and cardiovascular, endocrine and immune systems. The biological signature of frailty can be seen in dysregulation of many metabolic pathways [85,86]. The aforementioned theories of aging may be explained, at least in part, by the damage generated by ROS and mediated by inflammations markers.

Oxidative stress is related to frailty by different pathways. In light of RNA oxidation, Liang and colleagues found that urinary 8-oxo-7,8-dihydro-2′-deoxyguanosine, as a recognized biomarker of RNA oxidation, was independently associated with frailty in elderly patients with cardiovascular disease, although the correlation was weak [87].

In a prospective multi-center observational study among patients with multi-morbidity, absolute telomere length (aTL) was shortened in response to enhanced oxidative stress [88]. It was seen as an increased activity of SOD and decreased total antioxidant activity against ROS. In contrast, in the study by Martínez-Ezquerro et al., only telomere length was found to contribute to the frailty phenotype, while the association between lipid peroxidation and telomere length was weak [89].

Work by Serviddio et al. showed that circulating inflammation and oxidative stress markers correlate to frailty. They found that TNF-alpha, oxidized glutathione (GSSG), MDA and 4-hydroxy-2,3-nonenal- protein plasma adducts were more significantly increased in a frail elderly population, defined by Fried’s criteria, than non-frail [90].

Tembo et al. conducted many studies concerning oxidative stress and inflammation in a frail population. They found a positive association between total antioxidant capacity and frailty, as well as for IL-6 and frailty before and after accounting for age and body mass index [91,92]. In other research, on patients with cerebral small vessel disease and cognitive frailty, an increased lipid peroxidation measured by MDA level and lower SOD activity were seen together with elevated inflammatory markers (CRP, IL-6, TNF-α) [93].

In the randomized PolSenior study, which investigated 4979 elderly participants from Poland, a relationship between markers of inflammation (IL6 and CRP) and frailty were shown [94]. What is more, Hammami et al. showed positive associations between frailty score and pro-inflammatory markers: CRP, IL-6, IL-8 and TNF-α [95]. Moreover, in this study, the relationship between CRP and frailty was still strong and significant after adjusting for age. Another work by Marcos-Pérez et al. established powerful and quantitative associations between CRP and IL-6 as an inflammatory biomarkers in frail older adults [96]. Additionally, Ribeiro and colleagues demonstrated that CRP was not only a marker of chronic inflammation, but might also participate as an age-related frailty biomarker [97]. In a meta-analysis by Mailliez et al., in addition to CRP, four other biomarkers (in both genders: vitamin D, albumin, and hemoglobin; in men: free testosterone) were confirmed to be associated with frailty [98].

While previous studies have focused on single biomarkers theoretically involved in the pathogenesis of frailty, the abnormalities in leukocytes as well as in inflammatory markers were also observed [99]. Moreover, in postmenopausal women, the prospective analysis showed an elevated fibrin turnover and fibrinolysis markers, which are typical for increased inflammation, as independent predictors of frailty [100]. Further results were found in an Italian population investigated by Landino et al. They investigated older participants of the InCHIANTI study in relation to proteomic data to discriminate individuals with frailty from those who were robust [101]. The InCHIANTI investigator tested the association of over one thousand plasma proteins with frailty using logistic regression adjusted for age at baseline and sex. They demonstrated, with a high degree of accuracy, that several plasma proteins were associated with frailty status. The proteins that were most discriminating the frailty syndrome were represented by proteins involved in cell growth pathways, inflammation and blood coagulation.

Other bridges between frailty, oxidative stress and inflammation go through gut dysbiosis. In a frail elderly population, a decreased *Firmicutes* to *Bacteroidetes* ratio was seen together with changed microbiota-derived metabolites resulted in increased oxidative stress and elevated inflammation [102]. Moreover, increased abundance of *Mogibacteriacee*, *Coriobacteriacee* and *Eggerthella* taxa was found among frail, chronic kidney disease patients [103]. Additionally, the *Mogibacteriaceae* bacteria positively correlated to inflammatory markers.

Diet and exercise may have an influence on frailty and, if well organized, prevent or delay the syndrome, albeit while not increasing the incidence of frailty. Kobayashi et al. found an effective strategy for frailty prevention. High dietary total antioxidant capacity and a diet rich with proteins were strongly inversely associated with the prevalence of frailty [104], while in the work conducted by Jayanama, a higher percentage of saturated fatty acids (SFA) intake was associated with both increased frailty and mortality. The effect of SFAs on mortality was evident across levels of frailty [105].

Thus, oxidative stress and inflammation may point to treating such patients with frailty syndrome by diet, exercise or medications. A double-blind randomized controlled trial conducted by Bo et al. proved that a diet rich with antioxidative vitamins and high whey proteins preserves muscle mass and strength and increases quality of life for weak older people [106]. An analysis of some randomized control trials indicated an important improvement in muscle mitochondria function in terms of oxidative stress after physical training in elderly adults [107].

## 5. Conclusions and Future Directions

Both frailty and metabolic syndromes lead to the following consequences: poorer response to physical and/or mental stressors, increased risk of hospitalization, adverse outcomes, institutionalization and premature death [108]. Different metabolic pathways discussed in this review demonstrated that a similar pathogenesis underlies the development of the metabolic as well as the frailty syndrome in the context of oxidative stress and acceleration of inflammation. The disturbance of these various metabolic processes on the cellular, tissue and organ level have demonstrated that the syndemia of the syndromes represents two faces of the same coin.

**Figure 2:**
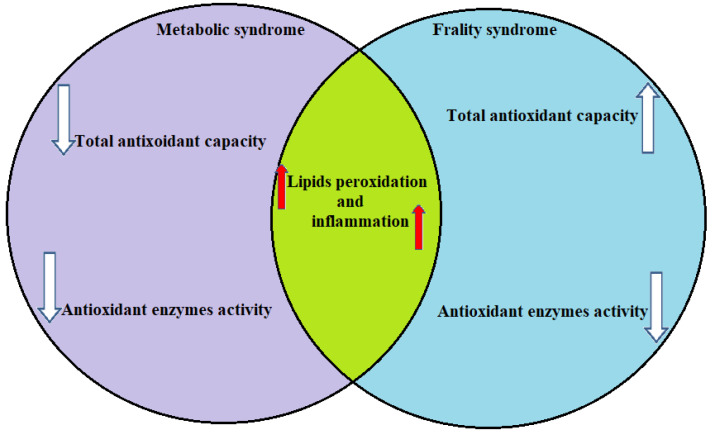
Changes in the inflammation and oxidative stress markers in both metabolic and frailty syndromes.

In the coming decades, due to a growing silver population, the syndemia of metabolic and frailty syndromes will increase. Figure 2 summarizes changes in the acceleration of the inflammation and increased oxidative stress in both metabolic and frailty syndromes. It shows similar faces of the pathomechanism in the syndromes. For this reason, we use the argument about two sides of the same coin.

At the same time, there are still many doubts and questions related to the syndemia. We already know that some components of frailty and metabolic syndromes may be delayed or even reversed [109,110]. However, some intervention showed next to no effect on mortality or other major outcomes [111]. Thus, we need more basic, translational and clinical research in the field of intervention of both metabolic and frailty syndrome.

Yet, higher numbers of both syndromes need the development of prevention programs for better longevity and to avoid premature deaths. The question is this: can syndromes in the elderly population be prevented globally? If so, prevention programs should be incorporated on the primary, secondary and tertiary levels. They should not only include the influence of important lifestyle risk factors such as diet and physical activity, but should also investigate sleep hygiene in relation to the syndemia as the primary prevention. Furthermore, the strategies for lifestyle changes and medications (which we did not explore) among the elderly population in the secondary and tertiary prevention of metabolic and frailty syndromes should be incorporated. What is more, already-known pathomechanisms should result in the development of preventive and therapeutic strategies to improve survival, increase longevity and reduce the number of co-morbidities as well as increase the quality of later life among elderly individuals.
